# 2022 Acknowledgment of *mSystems Ad Hoc* Reviewers

**DOI:** 10.1128/msystems.01097-22

**Published:** 2022-12-20

**Authors:** Jack A. Gilbert

**Affiliations:** a UC San Diego School of Medicine, La Jolla, California, USA

## EDITORIAL

Every year I write a brief statement of thanks for the hundreds to thousands of reviewers that make peer review possible at *mSystems*. Peer reviewing is a complex and often thankless task. The editors at *mSystems* are all specialists in their fields, but they are also all peer reviewers and authors. Having an author, reviewer, and editor all in the same brain can often create cognitive dissonance, as I have mentioned previously (https://doi.org/10.1128/mSystems.00797-19). However, all of us are committed to providing robust assessment, through constructive feedback, for every paper that we send out for peer review. Over the last 3 years, it has become increasingly hard to find reviewers willing to perform peer review. While I have no hard data as to why this trend is occurring, like most things, I blame it on the pandemic. However, there has also been a concomitant maturing of many different microbiome-focused journals, which has taxed a relatively small group of researchers, who are often asked to review many different manuscripts simultaneously. I routinely receive 10 to 15 peer-review requests per week. Obviously, no one has the time to assess anything rigorously with that kind of workload, so I end up saying “no”—a lot! How do I choose which ones I say yes to? Well, in the last 2 to 3 years I have been focusing on performing peer review only for society journals. The American Society for Microbiology, Applied Microbiology International, International Society for Microbial Ecology, and many others provide substantial resources to their membership that are in part supported by revenues from the journals. Therefore, by supporting the journals as an editor, reviewer, or an author, you are generating revenue that provides support for your scientific community, and maybe even yourself. What could be better? Thank you to all the reviewers who made peer review at *mSystems* such a success this last year, and thank you for supporting ASM and your community.

Ruth Chingcuangco Abanador
Ghulam Abbas
Wafaa Abd El-Ghany
Mahmoud M. Abdelsattar
Luis A. Actis
Zaky Adam
Beth M. Adamowicz
Oehmen Adrian
Innocent Afeke
Livnat Afriat-Jurnou
Herve Agaisse
Ashwani Aggarwal
Surya D. Aggarwal
Stephen Dela Ahator
Din Ahmad Ud
Tae-Hyuk Ahn
Lianzhong Ai
Sulaiman M. Alajel
M. Tauqeer Alam
Begum Alaybeyoglu
Virginia Helena Albarracín
Muhammad Ali
Celeste Allaband
Jonathan Allen
Richard C. Allen
Charlotte J. Alster
Hassan M. Al-Tameemi
Lauren Victoria Alteio
Emrah Altindis
John Alverdy
Dani Amor
Shivanthi Anandan
Mark T. Anderson
Matthew Zack Anderson
Michael Anderson
Angel Andrade Torres
Julien Andreani
Cristina Andrés-Barrao
Haike Antelmann
Vijay C. Antharam
Muzaffer Arikan
Jean Armengaud
Courtney R. Armour
Ravi P. Arya
Adrien Assie
Jennifer M. Auchtung
Lucas Auer
Sandrine Auger
Frank O. Aylward
Derya Aytan-Aktug
Sheyda Azimi
Reha O. Azizoglu
Jin-Woo Bae
Harsh Bais
Jonathon L. Baker
Scott E. Baker
Vimal Balasubramanian
Areen Banerjee
Kalins Bannerjee
Matthieu Barbier
Roman Alfredo Barco
Lars Barquist
Franco Basile
Ashley C. Bateman
Buzz Baum
Andreas J. Bäumler
Clifford J. Beall
Pascale B. Beauregard
Ashley E. Beck
Christine Beemelmanns
Terrence Bell
Daniel Bellieny-Rabelo
Michael Benisrael
Mariana Benitez
Trude Bennett
Fabienne Benz
Anne-Sophie Bergot
Anna Bernasconi
Anthony Bertagnolli
Martin Beukema
Mohini Bhattacharya
Derek Martens Bickhart
Joseph Bielawski
Jordan E. Bisanz
Pradeep Bist
Nidhan K. Biswas
Ryan Andrew Blaustein
Ran Blekhman
Mark Blenner
Samuel J. Bloomfield
Louis-Marie Bobay
Nick Bohmann
David Bolam
Luis M. Bolaños
Alte Bones
Joanna-Lynn C. Borgogna
Chiara Borsetto
Keith Bouma-Gregson
Travis Bourret
Cara C. Boutte
Robert Bowers
Patrick H. Bradley
Timothy S. Breton
Corinna Breusing
Sylvain Brisse
Amanda M. V. Brown
Bianca Regina Palmer Brown
Chris M. Brown
Allen Browne
Kirsteen Browning
Vincent Bruno
Gregory A. Buck
Michal Bukowski
Aaron Burberry
Alejandro Buschmann
Susheel Bhanu Busi
Yuming Cai
Douglas R. Call
Benjamin J. Callahan
Andrew Camilli
François-Xavier Campbell-Valois
Zoe Cardon
Carlo R. Carere
Allison F. Carey
Jeffrey Carey
Michael Carlson
Tyler J. Carrier
Catherine Carrillo
Santiago Castillo-Ramírez
Lindsay J. Caverly
Yunrong Chai
Dipshikha Chakravortty
Apostolos Chalkis
Rishi Chanderraj
Hao-Xun Chang
Benoit Chassaing
Kausik Chattopadhyay
Narendrakumar Chaudhari
Lianqiang Che
Tanya Cheeke
Ding-Qiang Chen
Huaihai Chen
Liang Chen
Songcan Chen
Wen-Hsiang Chen
Shu Cheng
Loo Wee Chia
Alexandra Chiaverini
Mariana Carmen Chifiriuc
Jason Chin
Byung-Kwan Cho
Davide Ciccarese
Gerard Clarke
Christine Clayton
Ana G. Cobián-Güemes
José Francisco Cobo Díaz
Maureen L. Coleman
James Collins
Fabian Commichau
Ian Frank Connerton
Lydia M. Contreras
Gregory M. Cook
Fernando Corrales
Claire Couch
Felipe H. Coutinho
Brett C. Covington
Don A. Cowan
John V. Cox
Keith A. Crandall
Alex Crits-Christoph
Jie Cui
Julia Cui
Ross Cunning
Chris Curtin
Tal Dagan
Zhongmin Dai
Ajai A. Dandekar
Kristen M. DeAngelis
Brian DeFelice
Patrick H. Degnan
Marcus de Goffau
Stephen De Lisle
Francesco Delogu
Ilenne Del Valle
Xin Deng
Yijie Deng
Laura De Nies
Mahesh S. Desai
Les Dethlefsen
Rae Devan
Neha Dhasmana
Rishu Dheer
Muhe Diao
Dulce Maria Diaz-Montaño
George C. Dicenzo
Christian Diener
Nicholas Dillon
Tatiana Dimitriu
Francisco Dionisio
Ray Dixon
Ulrich Dobrindt
Alex Dornburg
Gavin M. Douglas
Richard G. Douglas
Simon L. Dove
Glen D’Souza
Rebbeca Duar
Caroline Dube
Leonard Dubois
Frank Duca
Breck A. Duerkop
Karine Dufresne
Peter F. Dunfield
Ashley M. Dungan
Janani Durairaj
Avishek Dutta
Jonathan Dworkin
David Dyer
Stefan Dyksma
Emily Ebel
Anna Edlund
Moamen Elmassry
Akintunde Emiola
Melinda Anne Engevik
Tobias Engl
Hannah E. Epstein
Madeleine Ernst
Oier Etxebeste
Jay D. Evans
Damien Eveillard
N. L. Fahrenfeld
Yanhua Fan
Alonso Favela
Nicolas Feau
Sara Federici
Michael J. Federle
Kelli Feeser
Jie Feng
Xiaowen Feng
Timothy G. Ferdelman
Gabriel da Rocha Fernandes
Maria-Luz Fernandez
Val Fernández-Lanza
Justyna Fert-Bober
Celio Fernando Figueiredo Angolini
Taicia Pacheco Fill
Helmut Fischer
Amaranta Focardi
Kristen Foust
Edward M. Fox
Paraskevi C. Fragkou
Gad Frankel
Kyle Frantz
Shiri Freilich
Ayari Fuentes Hernández
Jed Fuhrman
Heather Fullerton
Cesare Furlanello
Chikara Furusawa
Giovanni Gallo
Maria Alessandra Gammone
Sukirth Ganesan
Raidah Gangji
Michael G. Ganzle
Xiang Gao
Xiaoyu Gao
Melanie G. Gareau
Matthew J. Gebert
Jennifer Geddes-Mcalister
Stephen J. Giovannoni
Catherine Girard
Alain P. Gobert
Filipa Godoy-Vitorino
Antonio Gonzalez Pena
Milena Gonzalo
David R. Goodlett
Tobias Goris
Claire L. Gorrie
Marcin Grabowicz
Matti Gralka
Peter L. Graumann
Michael Jeffrey Gray
Christen Grettenberger
Anne Grove
Alyssa Grube
Carsten Grupstra
Maria-Eugenia Guazzaroni
Eric Guédon
Sohini Guha
Fengbiao Guo
Lanping Guo
Vinod Gupta
Elaine M. Haase
Daniel H. Haft
Tatsuro Hagi
Aria S. Hahn
Matthias Hahn
Yang Hai
Sarah Haines
Larry J. Halverson
Joshua Hamm
Brian K. Hammer
Jens Andre Hammerl
Leendert W. Hamoen
Nancy D. Hanson
Jianjun Hao
Yanbin Hao
Janani Hariharan
Matthew J. Harke
Joshua Harrison
Erica M. Hartmann
Nur A. Hasan
Christiane Hassenrück
Roland Hatzenpichler 
Juliette Hayer
Anna Hayes
Bridget Hegarty
Johann Heider
Caitlin Heil
Jack A. Heinemann
Noelle Held
Véronique Helfer
Nicholas C. K. Heng
Carly Henkel
Michael Henson
Melissa M. Herbst-Kralovetz
Aaron D. Hernday
Rachel Hestrin
Robert L. Hettich
Teppo Hiltunen
Chris Hine
Ying-Ning Ho
Wouter D. Hoff
Casandra L. Hoffman
William Hofstadter
Deborah A. Hogan
James F. Holden
Suzie Hoops
Julie Horvath
Paul A. Hoskisson
Alice Hotopp
Xiaoyu Hu
Chao-Li Huang
En Huang
David Andrew-Essman Hufnagel
Wenwen Huo
Valentina Hurtado-Mccormick
Douglas L. Huseby
Filip Husnik
Fatima Hussain
Kevin Hybiske
S. Hyodo
Francesco Imperi
Keiichi Inaoue
Ikbal Agah Ince
Richard E. Isaacson
Elliot Walter Jackson
Scott A. Jackson
William R. Jacobs
Carsten Suhr Jacobsen
Cholsoon Jang
Jayanth Jawahar
Francis E. Jenney
Zhongjun Jia
Hongchen Jiang
Juquan Jiang
Sizun Jiang
Yong Jiang
Ziyun Jiang
Shuo Jiao
Mona Johannessen
Anders Johansson
Zackary Johnson
Eric Jones
Stuart Jones
Peter Jorth
Jayadev Joshi
Jyoti Joshi
Ari Jumpponen
Sheryl Justice
Lindsay Kalan
Marina G. Kalyuzhnaya
Brandi Kamermans
Vinayak Kapatral
Vera Karlicic
Lisa Karstens
Kazuyuki Kasahara
Takao Kasuga
Kathryn Kauffman
Shanlin Ke
Christina A. Kellogg
Patrick Kemmeren
Michelle A. Kennedy
Jannigje Gerdien Kers
Shahrbanoo Keshavarz Azizi Raftar
Chetan Keswani
Brandon Kieft
Kristopher Kieft
Min-Soo Kim
Taegyu Kim
Jeffrey Alan Kimbrel
Claas Kirchchelle
Kwan Soo Ko
Hitoshi Komatsuzawa
Weidong Kong
Konstantinos T. Konstantinidis
Bon-Sang Koo
Christian Kost
Aleksandar D. Kostic
Károly Kovács
Sascha Krause
Jens Kreth
Nicolas Krink
Arianna Krinos
Lee Kroos
Lee R. Krumholz
Anand Kumar
Rolf Kümmerli
Benoit Kunath
Jahnavi Kurasam
Viola Kurm
Kyohei Kuroda
Kirsten Küsel
Simon Labarthe
Steve Labrie
Leo Lahti
Regina Lamendella
Lefu Lan
Zachary Landry
Yelena Lapidot
Gisele Lapointe
Beatrice Laroche
Christian Lauber
Donald W. Lawrence
Christopher E. Lawson
Chih-Ying Lay
Francois Lebreton
Angelica Lebron
Alice Ledda
Charles Kai-Wu Lee
Eun Yeol Lee
Grace C. Lee
Jean-Luc Legras
Owen P. Leiser
Danielle G. Lemay
Cammie F. Lesser
Petra Anne Levin
Janina P. Lewis
Dan Li
Fu-Li Li
Fuyong Li
Meng Li
Xian-Zhi Li
Xiaogang Li
Zi-Bo Li
Guanxiang Liang
Chen Liao
Josie Libertucci
Ian Dennis Edmund Alan Lidbury
Tami Lieberman
Virginia Lioy
Bin Liu
Gang Liu
Jia Liu
Jinxin Liu
Xingyin Liu
Ya-Jun Liu
Yu-Rong Liu
Yannan Liu
James Locke
Brett Loman
Allison Lopatkin
Stilianos Louca
Brian Michael Luna
Justin Lund
Elaine Luo
Yuheng Luo
Zhao-Qing Luo
Jonathan Lynch
Meinan Lyu
Jing Mei Ma
Liyuan Ma
Mary Machado
Roderick I. Mackie
D. Mitch Magee
Jennifer Mahony
Rabindra Kumar Mandal
Subhrangshu Mandal
Amee Manges
Michael Manhart
Shrikant Subhash Mantri
Alejandro Manzano-Marín
Ramona Marasco
Maria L. Marco
Pierre Marijon
Igor Marín de Mas
Jessica L. Mark Welch
Clarisse (Lisa) Marotz
William Martin
José Luis Martínez
Esteban Martínez-García
Andreza F. Martins
Adam Martiny
Cristina Marzachì
Jen Matthews
Florent Mazel
Bonita McCuaig
Joy A. McKenna
Katelyn McNair
Alan McNally
Stephen J. McSorley
Daniel R. Mende
Alessio Mengoni
Sebastian Menke
Hayden Metsky
Eisha Mhatre
Luciana Migliore
Andrew D. Millard
Yusuke Minato
Jeremiah J. Minich
Sara Mitri
Jennifer M. Mobberley
Omkar Satyavan Mohite
Mariano A. Molina
Karen M. Moll
Denise M. Monack
Shirin Moossavi
Daniela Morales-Sanchez
Nancy A. Moran
Karen Moreau
Christopher E. Morgan
Morigen Morigen
Jennifer L. Morrow
Jamie Morton
Benjamin Douglas Moser
Luis Mota-Bravo
Thabiso Eric Motaung
Iwona Mruk
Ryan Sean Mueller
Arunika Mukhopadhaya
Sunil Mundra
Brian T. Murphy
Mario E. Muscarella
Vivek K. Mutalik
Jillian Myers
Jatin Nagpal
Dipti D. Nayak
Stephen Nayfach
Andrew L. Neal
Brittany Needham
William C. Nelson
Trang T. H. Nguyen
Jens Nielsen
Vincent Nieto
Yosuke Nishimura
Cecilia Noecker
Juan Nogales
Teresa Nogueira
Daniel R. Noguera
Noelle Noyes
Dele Ogunremi
Mitsuo Ogura
Norio Ohmagari
Kinji Ohno
Anil Ojha
Angela Oliverio
Elin Org
William D. Orsi
Orla O’Sullivan
Paul W. O'Toole
Hong-Yu Ou
Anne-Marie Overstreet
Mustafa Özçam
Alan Roberto Pacheco
Cátia Pacífico
Jon E. Paczkowski
Melinda Paholcsek
Sepideh Pakpour
Marton Palatinszky
Robert J. Palmer
Meichen Pan
Gaurav Pandharikar
Jason A. Papin
Tanya Parish
Tansol Park
Elizabeth Parkinson
D. Joshua Parris
Edoardo Pasolli
Alessandro Passera
Nastassia V. Patin
Kathryn A. Patras
Amaury Payelleville
José Penades
Luciana Pereira
Araceli Perez-Lopez
Everett C. Pesci
Rita de Cassia Pessotti
Jason M. Peters
Scott Peterson
Hrvoje Petkovic
Catherine Ann Pfister
Cécile Philippe
Aurore Picot
Flavia Pinzari
Mircea Podar
Claudia Pogoreutz
Phillip B. Pope
Davide Porcellato
Jan Postberg
Bradley E. Poulsen
Akbar Adjie Pratama
Eva C. Preisner
David Price
G. W. Price
John G. Purdy
Leah Pyter
Fahd Qadir
Weigang Qiu
John Quale
Robert Andrew Quinn
Lilliana Radoshevich
Sadequr Rahman
Tracy Lyn Raivio
Geeta Ram
Gordon Ramage
Thandavarayan Ramamurthy
Karthik Raman
Kelly Ramirez
David Rasko
Tamar Ratishvili
Christoph Ratzke
Samiksha Raut
Kasie Raymann
Timothy D. Read
María Rebolleda Gómez
Nicole Redvers
Andreas Reisner
Taylor Elaine Reiter
Linta Reji
Ze Ren
Alejandro Reyes
Todd B. Reynolds
François Ribalet
Charlie Rice
Jason M. Ridlon
Alice Risely
John Roberts
Francisco Rodriguez-Valera
Roderich Roemhild
Geraint B. Rogers
Rebekah Rogers
John R. Rohde
Veronica Roman-Reyna
David Romero
Søren Rosendahl
Corinna Ross
Charlène Roussel
Denis Roy
Daniel Rozen
Albane Ruaud
Peter Rubbens
John Rubinstein
Lorena Ruiz
Alistair Russell
Jason W. Sahl
Saaz Sakrikar
Álvaro San Millan
Magdalena San Roman
Tasha Marie Santiago-Rodriguez
Alyson E. Santoro
Rumakanta Sapkota
Guillaume Sarrabayrouse
Mariana Gabriela Sartorio
Goor Sasson
Matthew J. Scarborough
Bernhard H. Schink
Dirk Schneider
Hannah Doris Schweitzer
Alison J. Scott
Nichollas E. Scott
Julie A. Segre
H. Steven Seifert
Jana Seifert
Yuji Sekiguchi
Alberto Severini
Heike Seybold
Lauren Marie Seyler
Vahid Shahrezaei
Migun Shakya
Hongmei Shang
Yongqi Shao
Zongze Shao
Jason W. Shapiro
Ashok Sharma
Saadlee Shehreen
Cody Sheik
Jiaxian Shen
Paul Sheridan
Zhou Jason Shi
Shota Shibasaki
Amanda Shore
Joshua D. Shrout
Weitao Shuai
Ella T. Sieradzki
Anna Simpson
Abhijeet Singh
Baneshwar Singh
Surender Singh
George Siopis
Clayton M. Small
Chuck Randall Smallwood
Michael Smanski
Paul Smith
Christian Sohlenkamp
Michael B. Sohn
Virtu Solano Collado
Kevin V. Solomon
Vincent Somerville
Bokai Song
Utkarsh Sood
Patrick Sorensen
Rogerio R. Sotelo-Mundo
Venky Soundararajan
Diana Zita Sousa
Barbara Spellerberg
Pierre Stallforth
Adi Stern
Nejc Stopnisek
Jorg Stulke
Jian-Qiang Su
Xiaoquan Su
Zhengchang Su
Smitha Sukumar
Anne O. Summers
Chaomin Sun
Hui-Zeng Sun
Jian Sun
Jun Sun
Mangesh Suryavanshi
Douglas Sweeney
Kazuhiro Takemoto
Chandni Talwar
Yinjie J. Tang
Gerald W. Tannock
Hannah Tavalire
Tegwin Taylor
Anna Taylor
Eva Teira
Benno Herman Ter Kuile
Janne Gesine Thöming
Vivek Thumbigere-Math
Xiaojun Tian
Curtis Tilves
Anna D. Tischler
Ananda Tiwari
Franklin Toapanta
Vittorio Tracanna
Emmanuel Treiner
Brian K. Trevelline
Gareth Trubl
Hein Tun
Tomasz Wojciech Turowski
Silvia Turroni
Vaibhav Upadhyay
Blake Ushijima
Balázs Vajna
Ariena H. C. van Bruggen
Justin J. J. van der Hooft
Janneke van de Wijgert
Simon vanVliet
Willem J. B. van Wamel
Jan-Willem Veening
Claire Veneault Fourrey
Marco Ventura
Ben Vezina
Pedro Vieira-Baptista
Sara Vieira Silva
Flora Julie Vincent
Joseph M. Vinetz
Marie-Joelle Virolle
Jay Vornhagen
Willem Waegeman
Irene Wagner-Dobler
Brett Wagner Mackenzie
David W. Waite
Steffen Waldherr
Aaron Walsh
Marina Resendes de Sousa Walther-Antonio
Hai-Hong Wang
Minggui Wang
Nancy Wang
Quanxi Wang
Wenjie Wang
Xueshan Wang
Yong Wang
Zhang Wang
Zhong Wang
Alex D. Washburne
Kenneth Wasmund
Samantha Che Waterworth
Thomas D. Watts
Shasta Webb
Lucy A. Weinert
Maggie Weinroth
Brian James Werth
Nicole Wheeler
Leann Whitney
Stefanie Widder
Lutz Wiehlmann
Claire E. Williams
Tomasz Wilmanski
Amanda Marie Wilson
Michael R. Wilson
Etthel Martha Windels
Nathan Wisnoski
Robyn J. Wright
Wei-Li Wu
Zhihong Xie
Yunhe Xu
Zhihui Xu
Jason H. Yang
Jun Yang
Liang Yang
Qian Yang
Yuyi Yang
Zhaomin Yang
Min Yap
Simon Ye
Weimin Ye
Avihu Yona
Nicholas D. Youngblut
Loubna Youssar
Hongwei D. Yu
Kaifan Yu
Richard Yu
Min Yue
Olivier Zablocki
Joseph P. Zackular
Arsalan H. Zaidi
Remi Zallot
Fabio Zanini
Lisa Zeigler Allen
Qinglu Zeng
Qixiao Zhai
Kateryna Zhalnina
Cong Zhang
Dao-Feng Zhang
Jie Zhang
Lei Zhang
Liangliang Zhang
Mingzi Zhang
Rong Zhang
Rui Zhang
Tianyu Zhang
Xiaobo Zhang
Yongjun Zhang
Zheng Zhang
Jiangchao Zhao
Jinbiao Zhao
Wenjing Zhao
Xilin Zhao
Beiwen Zheng
Hao Zheng
Hong Zheng
Jianqiu Zheng
Xiang Zheng
Xueyun Zheng
Xiang Zhong
Zengtao Zhong
Zhi-Ping Zhong
Xingang Zhou
Yu Zhou
Baoli Zhu
Lei Zhu
Lifeng Zhu
Long-Ji Zhu
Qiyun Zhu
Wenhan Zhu
Yongzhang Zhu
Ryan M. Ziels
Nadine Ziemert
Michael Zimmermann
Franz Georg Zingl
Erik R. Zinser
Moreno Zolfo
Qiyun Zu
Bang 冯

